# Inhibition of Prostaglandin Transporter (PGT) Promotes Perfusion and Vascularization and Accelerates Wound Healing in Non-Diabetic and Diabetic Rats

**DOI:** 10.1371/journal.pone.0133615

**Published:** 2015-07-31

**Authors:** Zhongbo Liu, Outhiriaradjou Benard, Mahrukh M. Syeda, Victor L. Schuster, Yuling Chi

**Affiliations:** 1 Department of Medicine, Albert Einstein College of Medicine, Bronx, New York, NY, United States of America; 2 Department of Physiology and Biophysics, Albert Einstein College of Medicine, Bronx, New York, NY, United States of America; University of Edinburgh, UNITED KINGDOM

## Abstract

Peripheral ischemia, resulting from diminished arterial flow and defective local vascularization, is one of the main causes of impaired wound healing in diabetes. Vasodilatory prostaglandins (PGs), including PGE_2_ and PGI_2_, regulate blood flow in peripheral tissues. PGs also stimulate angiogenesis by inducing vascular endothelial growth factor. However, PG levels are reduced in diabetes mainly due to enhanced degradation. We hypothesized that inhibition of the prostaglandin transporter (PGT) (SLCO2A1), which mediates the degradation of PGs, would increase blood flow and stimulate vascularization, thereby mitigating peripheral ischemia and accelerating wound healing in diabetes. Here we report that inhibiting PGT with intravenously injected PGT inhibitor, T26A, increased blood flow in ischemic hind limbs created in non-diabetic rats and streptozotocin induced diabetic rats. Systemic, or combined with topical, T26A accelerated closure of cutaneous wounds. Immunohistochemical examination revealed that inhibition of PGT enhanced vascularization (marked by larger numbers of vessels formed by CD34+ cells), and accelerated re-epithelialization of cutaneous wounds. In cultured primary human bone marrow CD34+ cells and human epidermal keratinocytes (HEKs) either inhibiting or silencing PGT increased migration in both cell lines. Thus PGT directly regulates mobilization of endothelial progenitor cells (EPCs) and HEKs, which could contribute to PGT-mediated vascularization and re-epithelialization. At the molecular level, systemic inhibition of PGT raised circulating PGE_2_. Taken together, our data demonstrate that PGT modulates arterial blood flow, mobilization of EPCs and HEKs, and vascularization and epithelialization in wound healing by regulating vasodilatory and pro-angiogenic PGs.

## Introduction

Diabetes-associated non-healing lower extremity wounds, including leg ulcers and foot ulcers, are major contributors to non-combat limb loss [1]. Impaired wound healing in diabetes is multi-factorial, including peripheral ischemia due to diminished arterial blood flow and defective local vascularization[2,3].

Blood flow from arteries mobilizes nutrients, progenitor cells, and other molecular mediators to peripheral tissues during wound healing, and is a prerequisite for mounting a successful repair response [4]. Endothelial progenitor cells (EPCs), mobilized by blood flow, support vascularization, which are essential for wound healing. In diabetes, occlusive peripheral arteries limit blood flow to distal tissues [5–8]. In addition, the endothelium is dysfunctional and EPCs are reduced [9,10], such that, at diabetic wound sites, these cells are incapable of properly forming vessels in a timely manner [11].

Prostaglandins (PGs), such as PGE_2_ and PGI_2_, are vasodilators, maintaining adequate blood flow to peripheral tissues[12–15]. PGE_2_ also promotes angiogenesis by inducing vascular endothelial growth factor (VEGF)[16–18]. Levels of PGE_2_ and PGI_2_ in the circulation are regulated by both synthesis and degradation. The latter is mediated by the prostaglandin transporter (PGT, SLCO2A1) in series with 15-OH PG dehydrogenase (15PGDH) [19]. We have found that global deletion or systemic inhibition of PGT raises PGE_2_ plasma levels in mice and rats[20–22], and that local application of a PGT inhibitor increases PGE_2_ at wound sites and accelerates cutaneous wound healing in both wild type and diabetic mice [18]. These studies led us to hypothesize that systemic inhibition of PGT would increase arterial blood flow to distal limbs and mitigate peripheral ischemia. Similarly, we hypothesized that topical application of a PGT inhibitor to wounds would increase vascularization at wound sites. Together, these systemic and local effects of PGT inhibition would accelerate wound healing. This study aimed to test these hypotheses by using streptozotocin (STZ)-induced diabetic rats and their non-diabetic controls.

## Materials and Methods

### Animals

Male Sprague Dawley rats of 200–250 g were purchased from Charles Rivers. STZ was injected intraperitoneally at a dose of 50 mg/Kg body weight, once daily, for 5 consecutive days. STZ rats that had a blood glucose level higher than 360 mg/dL were selected for experiments. All experimental procedures were approved by and performed in compliance with the guidelines of the Institutional Animal Care and Use Committee (IACUC) at Albert Einstein College of Medicine. All surgery procedures were conducted while animals were under continuous anesthesia with 2.5% isoflurane. For acute limb ischemia experiments lasting for 4–8 hours, animals were sacrificed immediately after the experiments were finished. For the cutaneous wound closure experiments, animals were sacrificed after all wounds closed. For histological examinations, at various time points during cutaneous wound healing rats were sacrificed right before tissue collections. The method of sacrifice is inhalation of carbon dioxide. Detailed procedures for each experiment are described in the following specific sections.

### Blood Flow

Blood flow in rat hind limbs and in cutaneous wounds that were created on the dorsa of rats was measured using a PeriScan PIM 3 Imaging System. All blood flow measurements were conducted while rats were anesthetized with 2.5% isoflurane and were placed on a heating pad at 37°C to maintain body temperature.

For blood flow measurements in the rat hind limbs, the femoral artery was isolated and partially occluded, and acute hind limb ischemia was established in one hind limb using an established tourniquet model of limb ischemia with some modification as follows[23–25]. Briefly, rats were continuously anesthetized with 2.5% isoflurane. Hair was removed from the hindquarters with a depilating cream, Nair from Walgreen. The femoral artery was exposed aseptically through a 5-mm incision and isolated from the femoral vein and nerve, then was ligated with No. 6.0 Prolene suture just above the bifurcation of the anterior epigastric and lateral caudal femoral arteries. It is important to note that the femoral vein stayed open. The tourniquet (No. 2 Prolene loop suture) was then passed underneath the femoral vessels to spare them and placed around the thigh as proximal as possible. Ischemia was achieved and controlled by tension on the tourniquet and clamping of the common femoral and superficial epigastric arteries. The other limb was used as control. Blood flow in the ischemic limb was measured before and after partial occlusion to ensure that ischemia was established. Tourniquet weight and clamping were adjusted periodically to ensure that blood flow was consistent when agents were absent or were washed out during experimental procedures. For administration of compounds intravenously the jugular vein was isolated and a polyethylene catheter (PE 50; 0.97 mm OK, 0.58 mm ID) was advanced into the right ventricle via the right jugular vein.

For blood flow measurements in the cutaneous wounds, circular full-thickness skin excisions of 10 mm diameter were created as described previously [18]. Agents were applied immediately after wounding and once every other day thereafter. Blood flow in the wound was measured immediately after wounding and every other day before agent re-application.

### Plasma PGE_2_ Measurement

To assess the effect of systemically injected T26A on PGE_2_ levels in the circulation in rats, 2 mL of blood was withdrawn from femoral artery 20 minutes after T26A injection via the jugular vein. Blood was immediately centrifuged at 5,000 rpm and 4°C for 15 minutes. Plasma was collected and kept at −80°C. PGE_2_ was measured using a PGE_2_ EIA kit from Cayman Chemical (Ann Arbor, MI, USA).

### Wound Closure

For purpose of monitoring wound closure and its associated cellular events, four 5 mm full-thickness cutaneous wounds were created on the dorsa of rats [18]. Vehicle or T26A was administered intraperitoneally (i.p.) immediately after wounding and thereafter once daily, until wounds were closed. In separate experiments, vehicle or T26A was applied both intraperitoneally once daily and topically once every other day immediately after wounding and thereafter, until wounds closed. Wounds were covered with fresh Tegaderm. Wound dressings were changed every other day after documentary digital photography. Wound sizes were analyzed using ImageJ. The open wound was defined as the unepithelialized area and the number of pixels was counted for quantification. Wound closure at experimental time points was calculated as percentage of initial wound area.

### Histological Examination of Wounds

At various time points, wounded rats were sacrificed for histological examination. Detailed method for processing and staining of cutaneous wound tissues was described previously [18]. Minor modification was that anti-CD34 polyclonal antibody (LifeSpan BioSciences, Seattle, WA, USA) interacting with rat tissues was used and the dilution was 1: 250.

### Cell Culture

Fresh primary human bone marrow CD34+ cells were obtained from AllCells (Alameda, CA, USA) and cultured according to the protocol from the supplier. Human epidermal keratinocytes (HEKs) were purchased from ScienCell (Carlsbad, CA, USA) and were cultured in serum free keratinocyte medium containing 1% keratinocyte growth supplement (ScienCell), 1% penicillin- streptomycin, and 5 mM glucose.

To choose optimal PGT siRNA, CD34+ cells or HEKs were seeded on 6-well plates. 24 hours later, when the confluency reached 40–60%, cells were transfected with 4 sets of siRNAs targeting PGT at various concentrations and GFP siRNA using RNAi Max transfection reagent (Life Technologies Corporation, Norwalk, CT, USA). GFP siRNA was used as a positive / negative control to determine transfection efficiency and the effect of silencing, without affecting PGT gene. Maximal silencing of PGT mRNA (80%) was achieved by transfecting PGT siRNA set 1 at concentration of 10 nM.

### Cell Migration Measurement

Cell migration was assessed by two methods, Cellular Wound Migration Assay and Transwell Assay, as described previously [18], with slight modification. For the Cellular Wound Migration Assay, 100,000 EPCs or HEKs were seeded onto 6-well plates and transfected with either control siRNA or PGT siRNA (Qiagen, Valencia, CA) 24 hours later. After cells reached 100% confluent, a gap in cells was made in the center of each well with a pipette tip of diameter of 1 mm. Cells were washed with PBS and incubated in medium. Phase contrast pictures were taken using a microscope (4 x objective) immediately after gaps were made, which was considered as 0 h time point. Immediately after picture taken cells were treated with or without 100 nM PGE_2_ or 5 μM T26A for 14 h (for EPCs) or 12 h (for HEKs) and pictures were taken again. The open area (not covered by cells) in the center of the well at 0 and 14 h (or 12 h) was determined using ImageJ. Closed area was calculated by subtracting the open area at 14 h (or 12 h) from the open area at 0 h. The percentage gap closure was calculated by dividing the closed area by the open area at 0 h.

For the Transwell Assay, 50,000 EPCs were seeded onto matrigel coated filters which were then inserted in 24-well plates. Twenty four hours later, cells were transfected with either control siRNA or PGT siRNA. Thirty six hours after transfection, cells were treated with or without 100 nM PGE_2_ or 5 μM T26A for 8 hours. Cells on the seeding (top) side of the filter were wiped with Q-tips. Remaining cells on the bottom side of filter were fixed with 4% paraformaldehyde at 25°C for 1 hour, and stained with 0.1% crystal violet for 1 hour. Cells on the bottom of the filter, which had migrated cells, were counted under a 10 x objective under a microscope.

### Cell Proliferation Assay

Cell proliferation was assessed using a cell proliferation ELISA with BrdU (Roche) according to the manufacturer’s protocol[26–28]. HEKs were seeded onto 96-well plate (10000 cells/well) in 100 ml serum free medium containing 1% keratinocyte growth supplement (ScienCell), 1% penicillin- streptomycin, 5 mM glucose. Twenty four hours later, cells were transfected with control or PGT siRNA. Twenty four hours after transfection, cells were treated with 100 nM PGE_2_ or 5 μM T26A for 2 days. During the last 2-hour of incubation, HEKs were pulse-labeled with 10 mM BrdU. BrdU incorporation was quantified by measurement with a Micro Plate Reader at 450 nm.

### Statistical analysis

Group measurements were expressed as average ± SEM. Comparisons between two groups were analyzed by Student’s t-test, or among multiple groups by ANOVA test, and p< 0.05 was considered significant.

## Results

### Peripheral Ischemia in Diabetes Is Associated with Reduced PGE_2_

**Fig 1:**
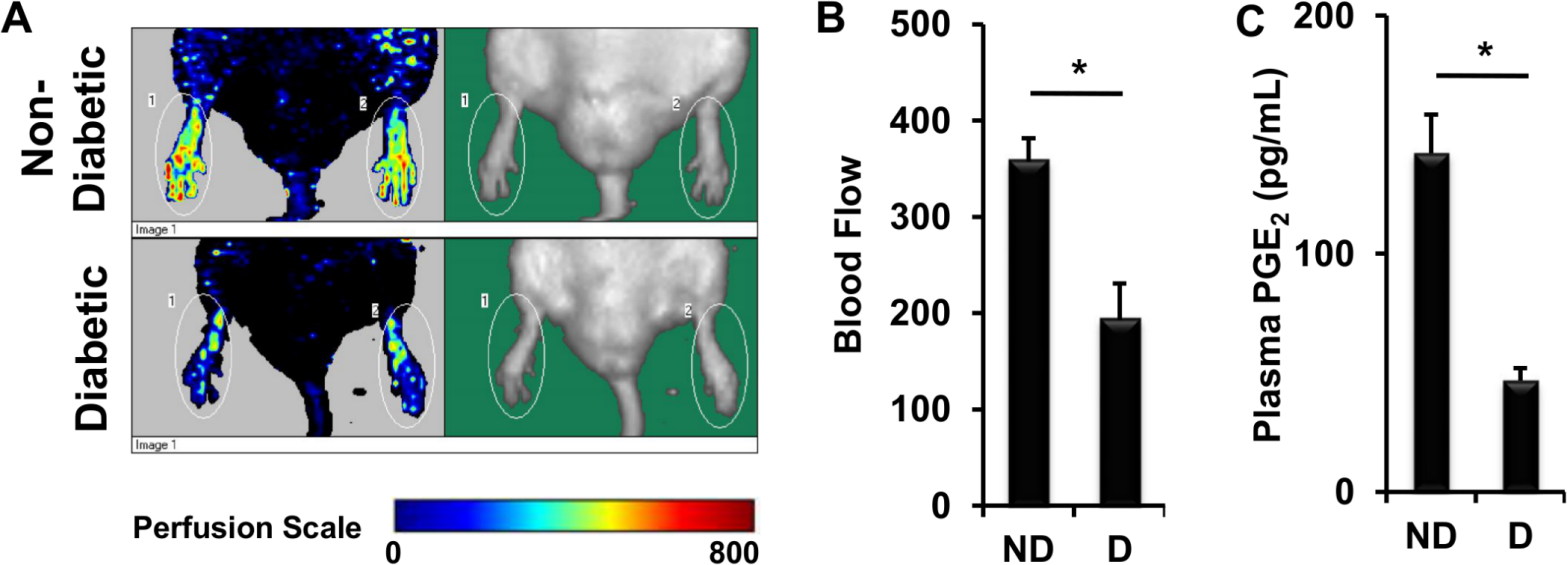
Peripheral Ischemia in Diabetes Is Associated with Reduced PGE_2_. (A) Images of representative blood flow in intact hind limbs of non-diabetic (ND) Sprague Dawley and STZ diabetic (D) rats. (B) Statistical analysis of blood flow in hind limbs. (C) Plasma PGE_2_. Values are average ± SEM (n = 5). *p < 0.05, p values were obtained by t-test.

Peripheral ischemia often occurs in diabetic patients [29,30]. To demonstrate this phenomena in animal model, we generated diabetic rats by injecting Sprague Dawley rats with STZ and measured blood flow in the hind limb of non-diabetic (ND) and diabetic (D) rats using a laser Doppler. Indeed, blood flow in hind limbs of STZ-induced diabetic rats was only half that of non-diabetic rats (Fig 1A and 1B). PGE_2_, as well as PGI_2_, are vasodilators and play important roles in regulation of blood flow [15]. Low PGE_2_ was reported in urine of diabetic rats [31]. Here we found that plasma PGE_2_ in diabetic rats was only about 30% that of non-diabetic rats (Fig 1C). These data suggest that peripheral ischemia in diabetes is accompanied with reduced PGE_2_.

### Systemic Inhibition of PGT Increases Perfusion to Distal Limb

**Fig 2:**
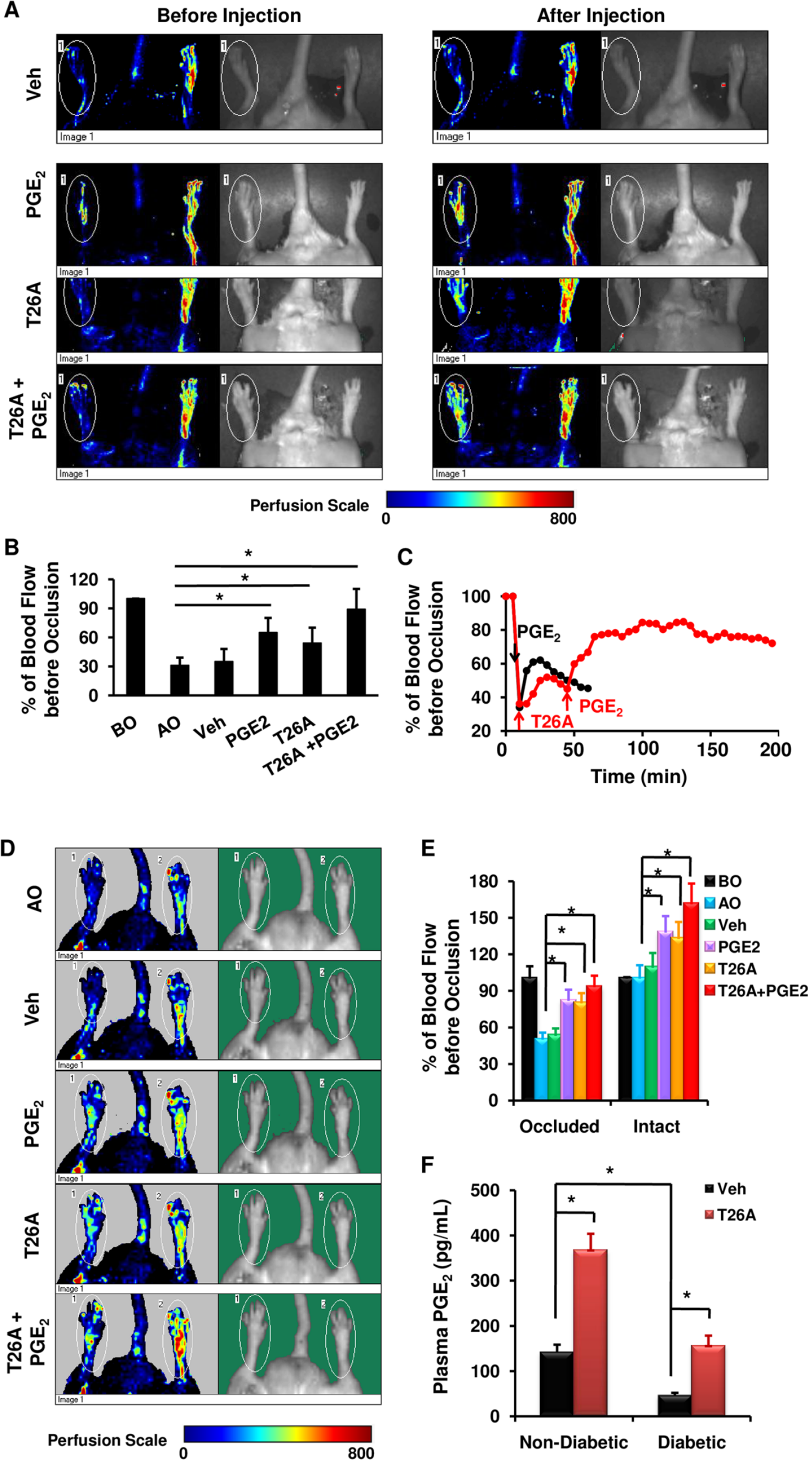
Systemic Inhibition of PGT Increases Perfusion to Distal Limb. (A) Images of representative blood flow in hind limbs of non-diabetic Sprague Dawley rats before and after various treatments. Left hind limb of each rat was partially occluded. 500 μL of vehicle (2% DMSO + 2% cremophor in water), or 10 μM PGE_2_, or 1.2 mM T26A was injected via jugular vein. (B) Statistical analysis of blood flow as percentage of blood flow before occlusion (BO). AO, after occlusion. (C) Representative pharmacodynamics of the effects of PGE_2_ and T26A on blood flow presented as percentage of blood flow before occlusion. (D) Images of representative blood flow in hind limbs of STZ diabetic rats before and after various treatments. Left hind limb of each rat was partially occluded. 500 μL of vehicle (2% DMSO + 2% cremophor in water), or 10 μM PGE_2_, or 1.2 mM T26A was injected via jugular vein. (E) Statistical analysis of blood flow as percentage of blood flow before occlusion (BO). Blood flow was measured using a PeriScan PIM 3. For all analyses of laser Doppler measurements the color scale was set at 0–800, and the intensity was set at 0.34. (F) Plasma PGE_2_. Values are average ± SEM (n = 5). *p < 0.05, p values were obtained by t-test for E and by ANOVA test for the rest.

We then asked whether exogenously applied PGE_2_ could rescue peripheral ischemia. To answer this question, we first used non-diabetic Sprague Dawley rats and created hind limb ischemia by partial occlusion of one of the hind limbs, while leaving the other intact. Vehicle or PGE_2_ was administered via jugular vein (S1A Fig). The average blood flow after occlusion was adjusted to be 30% of the value before occlusion (Fig 2A and 2B). Systemic PGE_2_ caused a 25% increase in blood flow in the reference limb (337 ± 24 (before administration) versus 421 ± 39 (after administration), n = 5, p < 0.05) (Fig 2A), demonstrating that systemic PGE_2_ can increase blood flow to peripheral tissues. In ischemic limbs, while the vehicle did not have significant effect, PGE_2_ doubled blood flow rates (Fig 2A and 2B), indicating that exogenous PGE_2_ can mitigate peripheral ischemia.

Our previous study showed that intravenously (i.v.) injected PGT inhibitor, T26A, in rats increased plasma PGE_2_ [21], indicating that i.v.T26A is bioavailable and systemic inhibition of PGT can effectively raise endogenous PGs. To test whether T26A had any effects on blood flow, we administered i.v. T26A to rats. Similar to PGE_2_, systemic T26A increased blood flow in the reference limb (Fig 1A) and doubled blood flow in ischemic limbs (Fig 2A and 2B). The combination of T26A and PGE_2_ tripled blood flow, bringing it almost to the level before occlusion (Fig 2A and 2B). The effects of PGE_2_ or T26A alone lasted for about 40 minutes (Fig 2C). However, pre-treatment with T26A prolonged the duration of PGE_2_ effects by more than 4-fold, consistent with an effect of T26A to prevent or significantly slow PGE_2_ metabolism (Fig 2C) [21]. Thus, systemic inhibition of PGT increases perfusion of peripheral tissues in ischemia.

To explore the clinical potential of T26A under diabetic conditions, we tested the effects of T26A and or PGE_2_ on blood flow in diabetic rats. In the intact hind limbs, treatment with PGE_2_ or T26A resulted in 30%–40% significant increase in blood flow. The combination of PGE_2_ and T26A increased blood flow to 160% that of untreated diabetic rats (Fig 2D and 2E), indicating that diabetic rats were responding to the treatments. Occlusion reduced blood flow to 50% of the level before occlusion (Fig 2D and 2E). PGE_2_, T26A or the combination returned blood flow to 80% of the level before occlusion (Fig 2D and 2E). Therefore, inhibition of PGT can mitigate ischemia in diabetic peripheral tissues.

To probe whether PGE_2_ was a molecular mediator of T26A effects on blood flow, we assessed PGE_2_ levels in the circulation of both non-diabetic and diabetic rats with or without T26A treatment. Intravenously injected T26A tripled plasma PGE_2_ in both non-diabetic and diabetic rats (Fig 2F), raising plasma PGE_2_ in diabetic rats to a level similar to that of non-diabetic rats (Fig 2F). These data suggest that T26A increases blood flow, probably, via raising endogenous PGE_2_.

### Inhibition of PGT Accelerates Cutaneous Wound Healing

**Fig 3:**
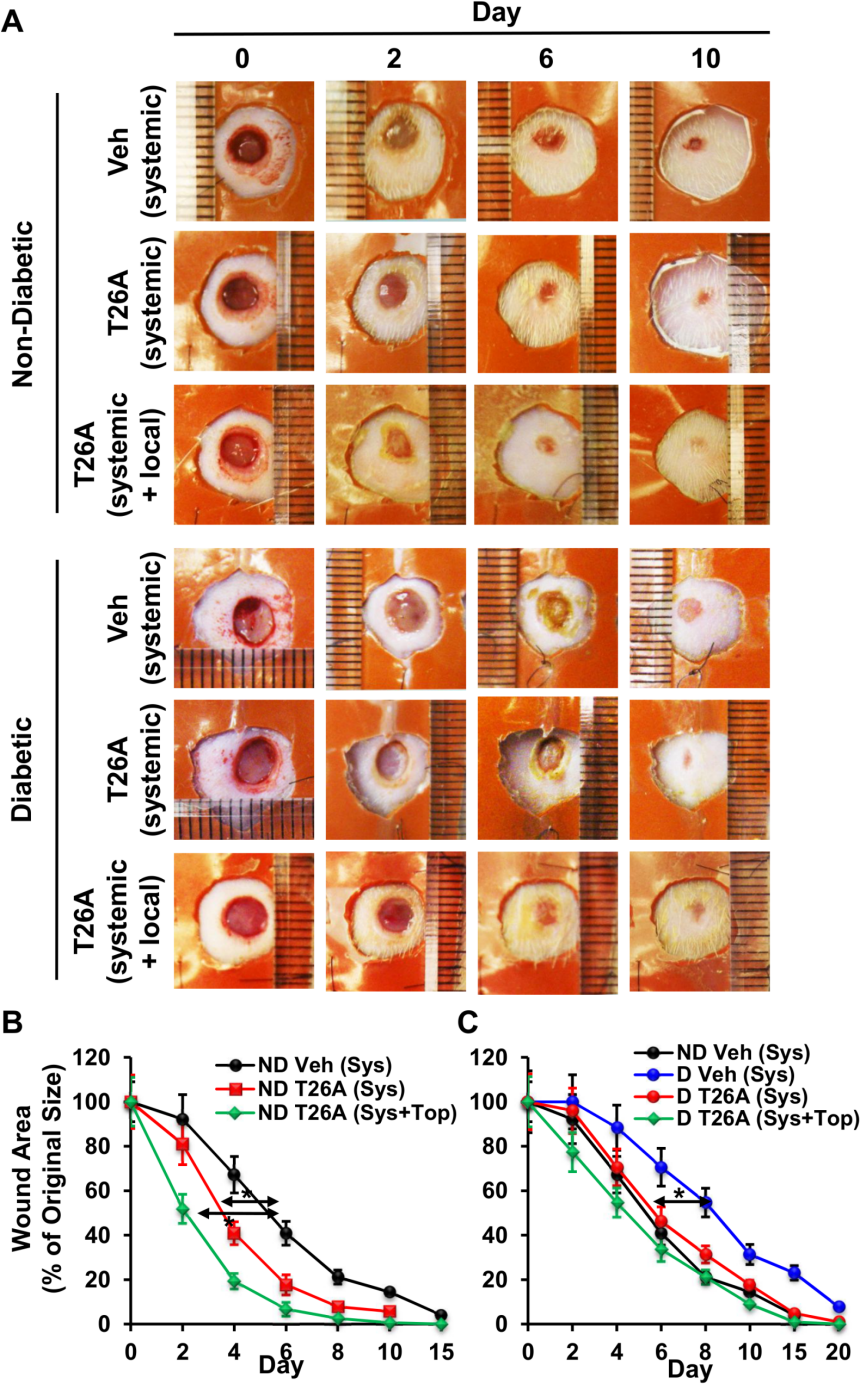
Inhibition of PGT Accelerates Wound Healing. (A) Photographs of representative cutaneous wounds in non-diabetic (ND) Sprague Dawley rats and STZ diabetic (D) rats on various days post-wounding. Four 5-mm cutaneous wounds were created on the opposite sides of the dorsa of rats. Vehicle (2% DMSO + 2% cremophor in water) or T26A was immediately applied either systemically alone or systemically plus topically. For systemic (Sys) application, 500 μL of vehicle or 1.2 mM T26A was injected intraperitoneally immediately after wounding and once daily thereafter. For topical (Top) application, 15 μL of vehicle or 2 mM T26A was applied to the wound immediately after wounding and every other day thereafter. (B, C) Wound closure rates of non-diabetic Sprague Dawley rats (B) or STZ diabetic rats (C) treated with PGT inhibitor (T26A) or vehicle. Values are average ± SEM (n = 5). *p < 0.05, p values were obtained by ANOVA test.

Adequate tissue perfusion is critical to cutaneous wound healing. To test whether enhanced peripheral perfusion by systemic inhibition of PGT could have any effects on cutaneous wound healing, we created cutaneous wounds on the dorsa of Sprague Dawley rats, administered i.p. T26A once daily, and measured wound size (see experimental design in S1B Fig). In the above blood flow experiments, T26A was administered via i.v. injection, because i.v. injection is a fast systemic route and rats did not need to be kept alive after the experiment. In this wound healing experiment, rats needed to be alive for the duration of wound healing and the suitable systemic administration was i.p. injection. I.p. T26A significantly shortened 50% wound closure time by 2 days and shortened complete wound closure time by 3–4 days (Fig 3A and 3B). Previously, we have shown that topical T26A accelerates cutaneous wound closure in mice [18], which led to the next set of experiments. In addition to systemic T26A, we applied T26A topically in the present rat model (S1B Fig). The combination of systemic and topical treatments resulted in further acceleration of wound healing over that of systemic T26A alone (Fig 3A and 3B).

While it took about 15 days for the wounds to close in untreated Sprague Dawley rats, it took 20 days for wounds to close in STZ diabetic rats (Fig 3A and 3C). Systemic T26A significantly shortened complete wound closure time in diabetic rats by 4 days. The combination of systemic and local T26A treatments further shortened wound closure time, bringing it similar to that of non-diabetic control rats (Fig 3A and 3C). These results demonstrate that systemic, or in combination with local, inhibition of PGT can mitigate impaired wound healing in diabetes.

### Inhibition of PGT Stimulates Vascularization

**Fig 4:**
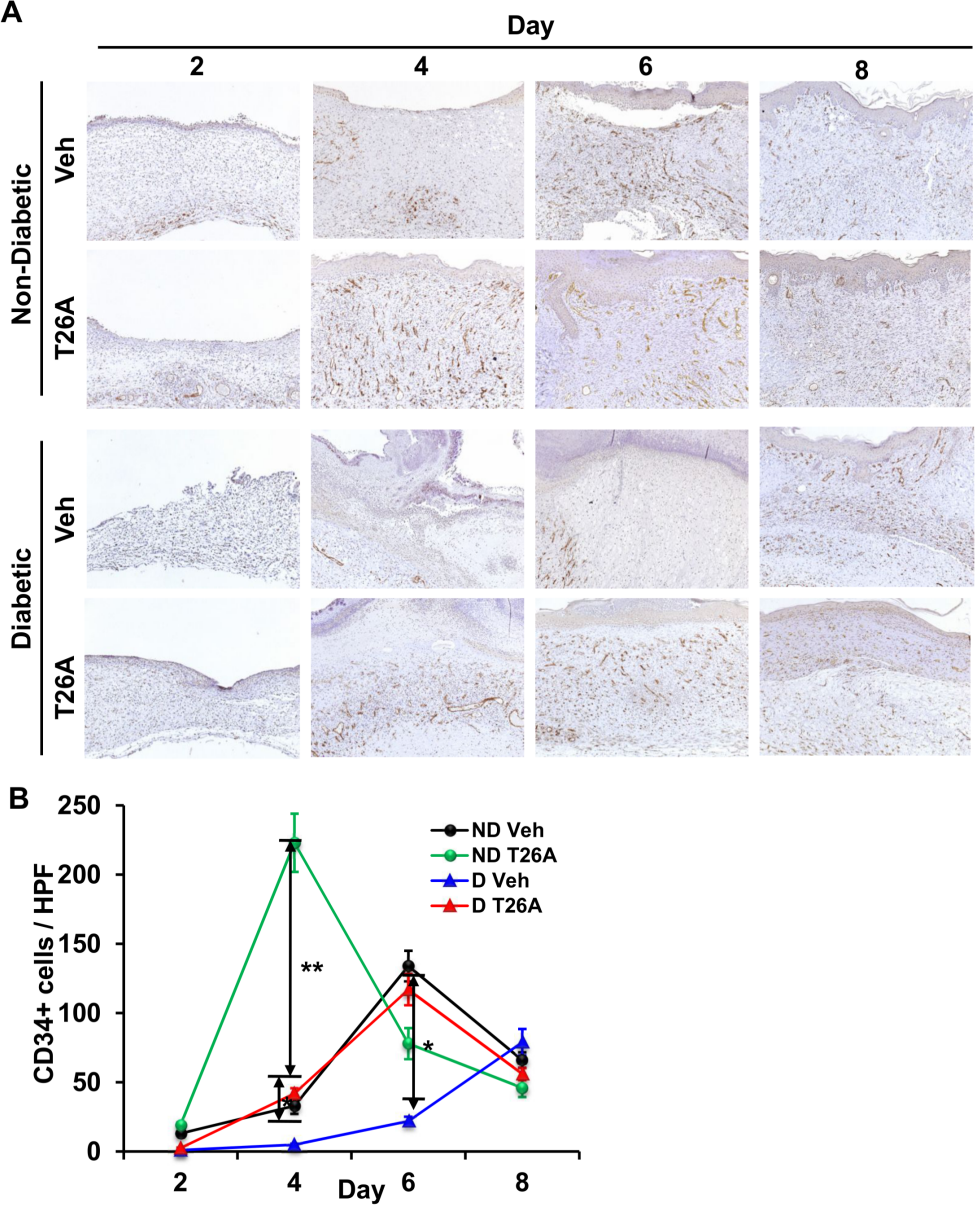
Inhibition of PGT Stimulates Vascularization. Representative images of CD34 (A) staining of cutaneous wounds of non-diabetic (ND) Sprague Dawley rats or STZ diabetic (D) rats. Rats were treated with both i.p. injected 500 μL of vehicle (2% DMSO + 2% cremophor in water) or 1.2 mM T26A, once daily, and topically applied 15 μL of vehicle or 2 mM T26A, once every other day. (B) Analysis of CD34+ cells. Numbers of CD34+ cells were counted in 5 random high power fields for each rat tissue. Five rats (per treatment) were used. Values are average ± SEM. *p < 0.05 or **p < 0.01, p values were obtained by ANOVA test.

Neovascularization is critical to wound healing. Histological examination revealed that rats treated with systemic and local T26A demonstrated neovascularization as early as day 2, as indicated by CD34 staining (Fig 4). T26A not only advanced the time point at which CD34+ cells peaked, from day 6 to day 4 in non-diabetic rats, but also doubled the amount of CD34+ vessels (Fig 4). After the peak time, amount of vessels started to decline as vessels reorganized.

Neovascularization was severely impaired in wounds of diabetic rats. At day 4 the moderate neovascularization observed in vehicle-treated non-diabetic rats was absent in diabetic rats. The amount of vessels in diabetic rats did not reach peak level until day 8, and consequently, the reorganization was delayed. In addition, the peak level of vessels in diabetic rats was only half of that in non-diabetic rats (Fig 4).

Systemic and local treatment with T26A significantly improved neovascularization in diabetic rats. T26A treatment resulted in modest CD34 reactivity in the wound bed at day 2, but a steady increase thereafter. The number of CD34+ cells in T26A-treated diabetic rats reached a peak at day 6 and started declining afterwards, indicating advanced reorganization, remodeling and healing of the wound. The peak level of vessels in T26A-treated diabetic rats at day 6 was 2-fold higher than that in vehicle treated diabetic rats at day 8 (Fig 4).

### PGT Regulates Endothelial Progenitor Cell Migration

**Fig 5:**
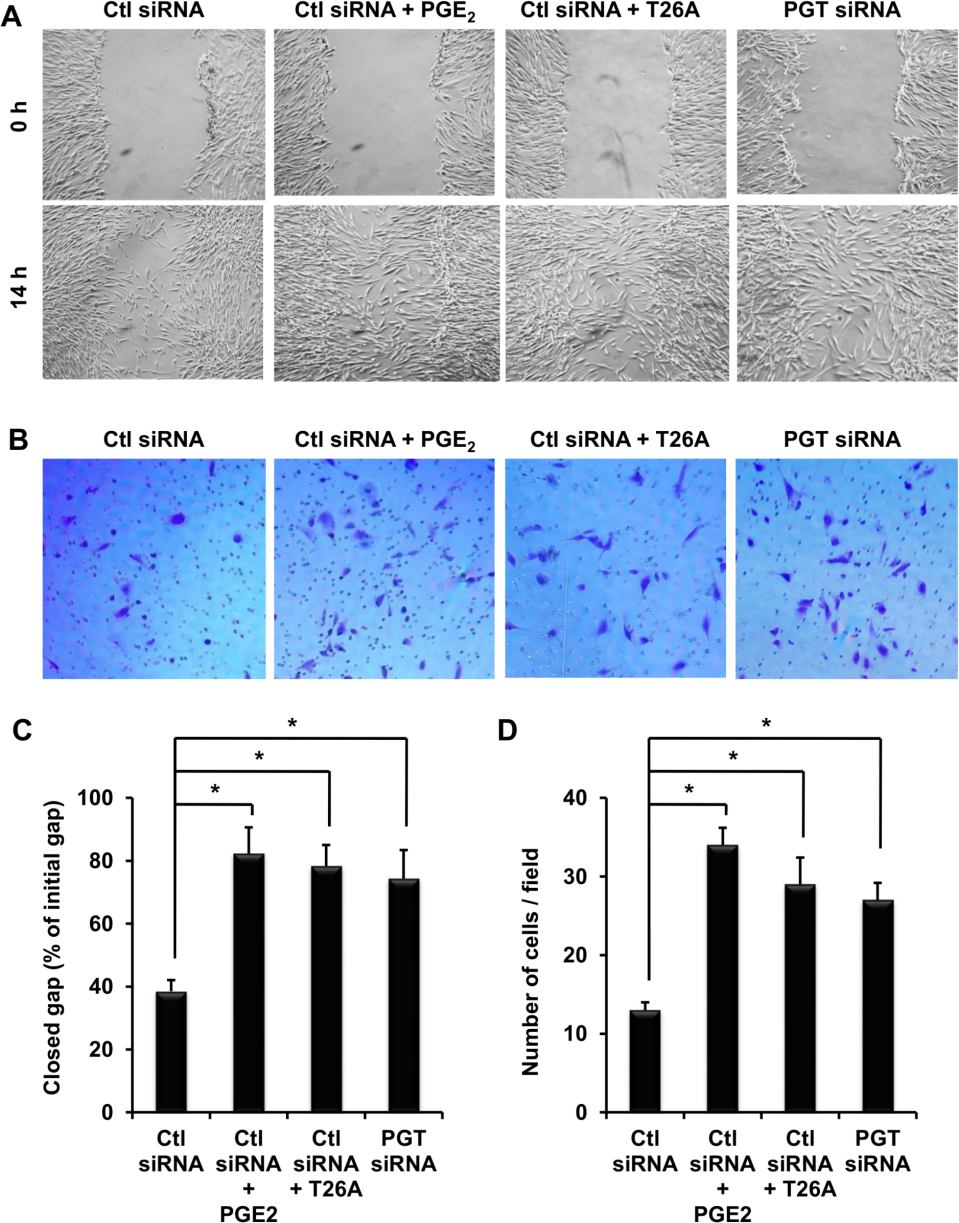
PGT Regulates Endothelial Progenitor Cell Migration. (A) Representative photographs of human bone marrow EPC wound migration. EPCs were seeded on 6-well plates and transfected with either control siRNA or PGT siRNA 24 hours later. After cells reached 100% confluent, gaps were created in the center of each well and pictures were taken. Immediately after picture taken, cells were treated with or without 100 nM PGE_2_ or 5 μM T26A for 14 hours and pictures were taken again. (B) Representative EPCs migrated through the filters. EPCs were seeded on matrigel coated filters which were then inserted in 24-well plates. Twenty four hours later, cells were transfected with either control siRNA or PGT siRNA. Thirty six hours after transfection, cells were treated with or without 100 nM PGE_2_ or 5 μM T26A for 8 hours. Cells on the seeding (top) side of the filter were wiped with Q-tips. Remaining cells on the bottom side of filter were fixed with 4% paraformaldehyde at 25°C for 1 hour, and stained with 0.1% crystal violet for 1 hour. Cells on the bottom of the filter, which had migrated cells, were counted with a 10 x objective under a microscope. (C) Analysis of EPC gap closure presented as percentage of closed gap to the initial gap. (D) Analysis of the transwell assay of EPCs migrated through the filters. These experiments were conducted for three rounds, each round in duplicate for each condition. Values are average ± SEM. *p < 0.05, p values were obtained by ANOVA test.

CD34+ cells are EPCs produced by bone marrow. Increased CD34+ cells at the wound site resulting from T26A treatment suggests that systemic inhibition of PGT stimulates migration of CD34+ cells traveling from the bone marrow to distal cutaneous wounds. To determine whether PGT directly modulates the mobility of EPCs, we performed migration assays in primary CD34+ cells from human bone marrow. After cells were transfected with siRNAs and subsequently reached 100% confluent, we created a 1-D gap and treated them with or without PGE_2_ or T26A for 14 hours. In wells transfected with control (Ctl) siRNA and without any treatment 38.5% of the gap closed (Fig 5A and 5C). PGE_2_ treatment accelerated gap closure to 82.3% at 14 hours. Treatment with T26A increased gap closure to 78.3%, similar to PGE_2_ treatment. To verify that T26A increased cell migration in response to T26A was due to inhibition of PGT, we transfected cells with PGT siRNA. Silencing PGT increased gap closure to 74.5% (Fig 5A and 5C). To confirm these results, we performed another migration assay, the transwell assay. While PGE_2_ increased the number of migrated cells through the filter by 2.5 fold, inhibiting or silencing PGT doubled that number (Fig 5B and 5D). The results obtained by these two migration assays consistently show that PGT directly regulates EPC migration and suppression of PGT enhances EPC mobility.

### Inhibition of PGT Stimulates Re-epithelialization by Enhancing Cell Migration and Proliferation

**Fig 6:**
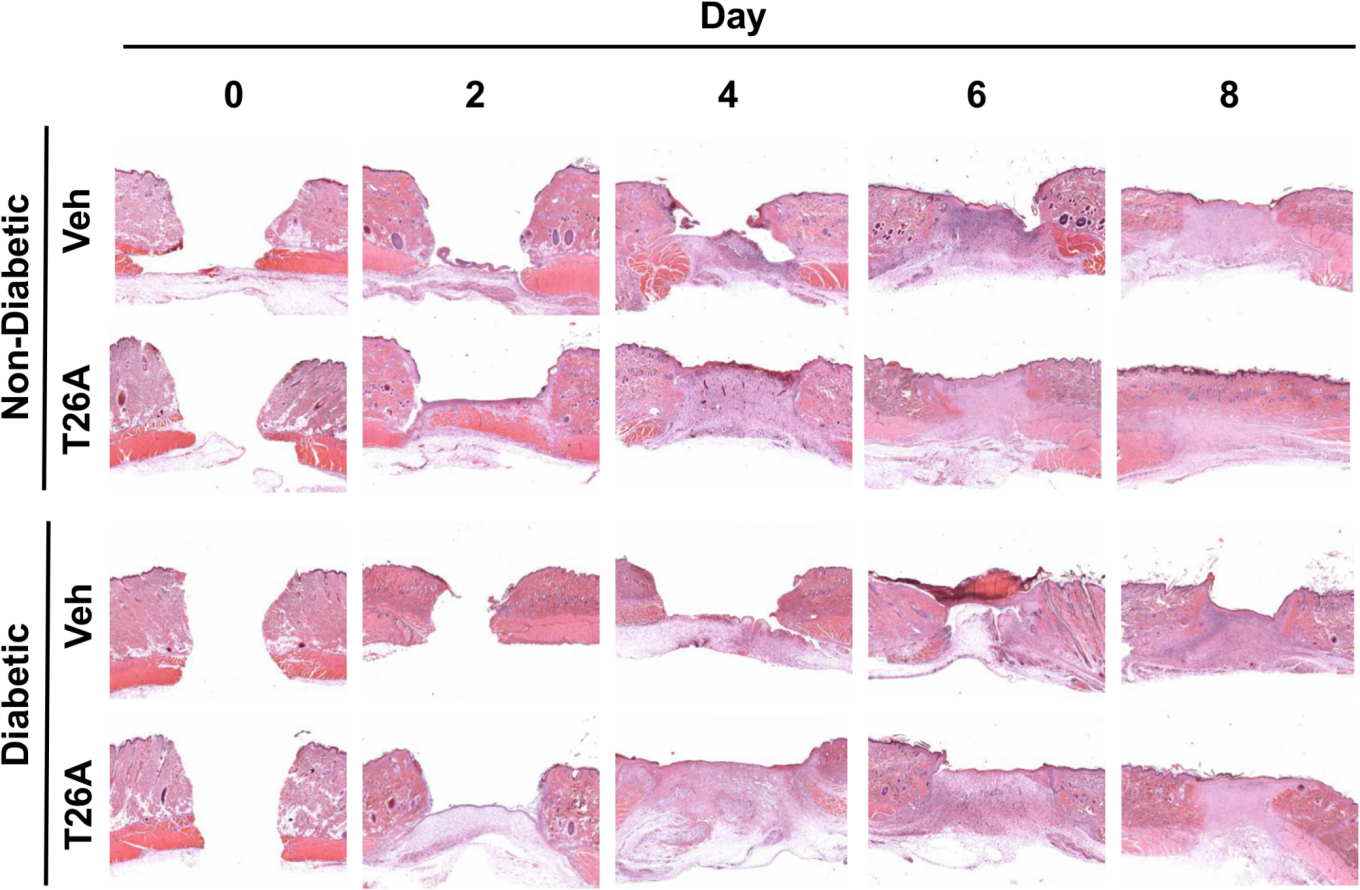
Inhibition of PGT Stimulates Re-epithelialization. Representative images of H&E (A) staining of cutaneous wounds of non-diabetic Sprague Dawley rats or STZ diabetic rats. Rats were treated with both i.p. injected 500 μL of vehicle (2% DMSO + 2% cremophor in water) or 1.2 mM T26A, once daily, and topically applied 15 μL of vehicle or 2 mM T26A, once every other day.

We performed additional histological evaluation of wound healing, including scoring the degree of epithelial coverage of the wound bed. At day 2, non-diabetic rats treated with topical and systemic T26A had greater re-epithelialization of the wound as compared to vehicle-treated rats (25% coverage vs. 10% coverage) (Fig 6). At days 4 and 6, there was 40% more papillary epithelial proliferation into the dermis in T26A-treated rats as compared to the vehicle-treated rats. At day 8, in T26A treated wounds, more than 90% of the gap was re-epithelialized over a smooth thin layer of granulation tissue, whereas in vehicle-treated wounds only 70% of the gap was re-epithelialized (Fig 6).

Epithelial migration over the wound was notably slower in wounds of diabetic rats compared to control rats, as re-epithelialization did not start until after day 2. At each time point, there was significantly less re-epithelialization in wounds of diabetic rats compared to control rats (Fig 6). In the re-epithelialized wounds, there was less papillary proliferation of the epithelium into the dermis (interpreted to be late development of hair follicles) in diabetic rats. Treatment with T26A accelerated re-epithelialization at each time point. At day 8, T26A treatment of wounds in diabetic rats resulted in 80–90% re-epithelialization and more papillary epithelial proliferation into the dermis compared to vehicle controls (Fig 6).

**Fig 7:**
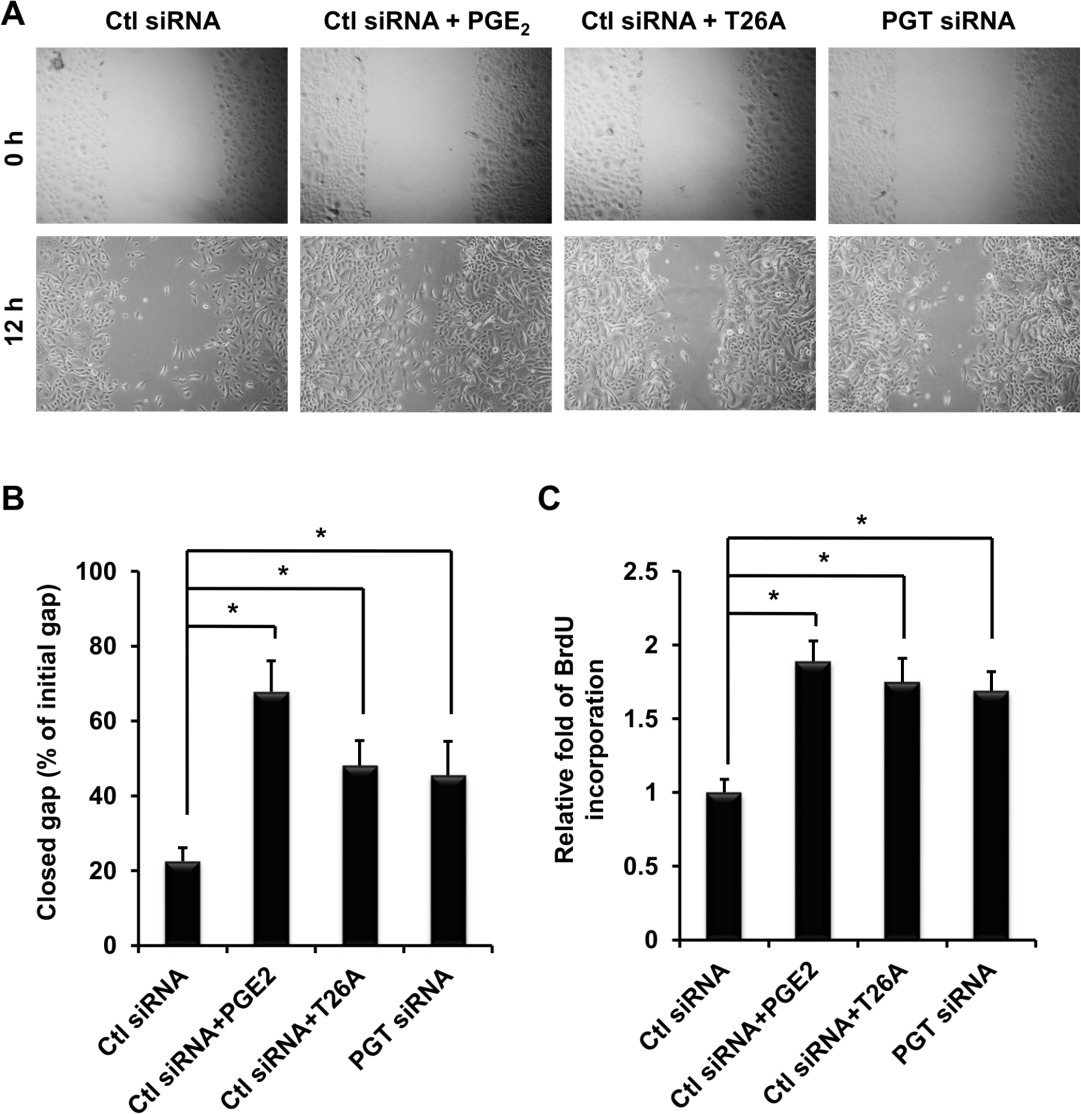
PGT Regulates Human Epidermal Keratinocytes Migration and Proliferation. Representative photographs of human epidermal keratinocytes (HEK) wound migration. HEKs were seeded on 6-well plates and transfected with either control siRNA or PGT siRNA 24 hours later. After cells reached 100% confluent, gaps were created in the center of each well and pictures were taken. Immediately after picture taken, cells were treated with or without 100 nM PGE_2_ or 5 μM T26A for 12 hours and pictures were taken again. (B) Analysis of HEK gap closure presented as percentage of closed gap to the initial gap. (C) Measurements of HEK proliferation by BrdU incorporation method. These experiments were conducted for three rounds, each round in duplicate for each condition Values are average ± SEM. *p < 0.05, p values were obtained by ANOVA test.

To confirm that PGT regulates epidermal cell migration, we conducted *in vitro* wound migration assay in HEKs in the presence or absence of PGE_2_ or T26. In wells transfected with control (Ctl) siRNA and without any treatment 22.5% of the gap closed 12 hours after gap creation (Fig 7A and 7B). PGE_2_ and T26A increased gap closure to 67.8% and 48.1%, respectively. To verify that T26A increased cell migration in response to T26A was due to inhibition of PGT, we transfected cells with PGT siRNA. Silencing PGT increased gap closure to 45.5% (Fig 7A and 7B).

Additionally, we assessed PGT modulation of HEKs proliferation. Fig 7C shows that PGE_2_ increased proliferation of HEKs by 1.89 fold as compared to control, in accordance with literature [32,33]. Both inhibition and silence of PGT significantly increased HEKs proliferation by 1.75 and 1.69 fold, respectively. These in vitro results show that PGT directly regulates HEK migration and proliferation.

### Inhibition of PGT Increases Perfusion of Cutaneous Wounds

**Fig 8:**
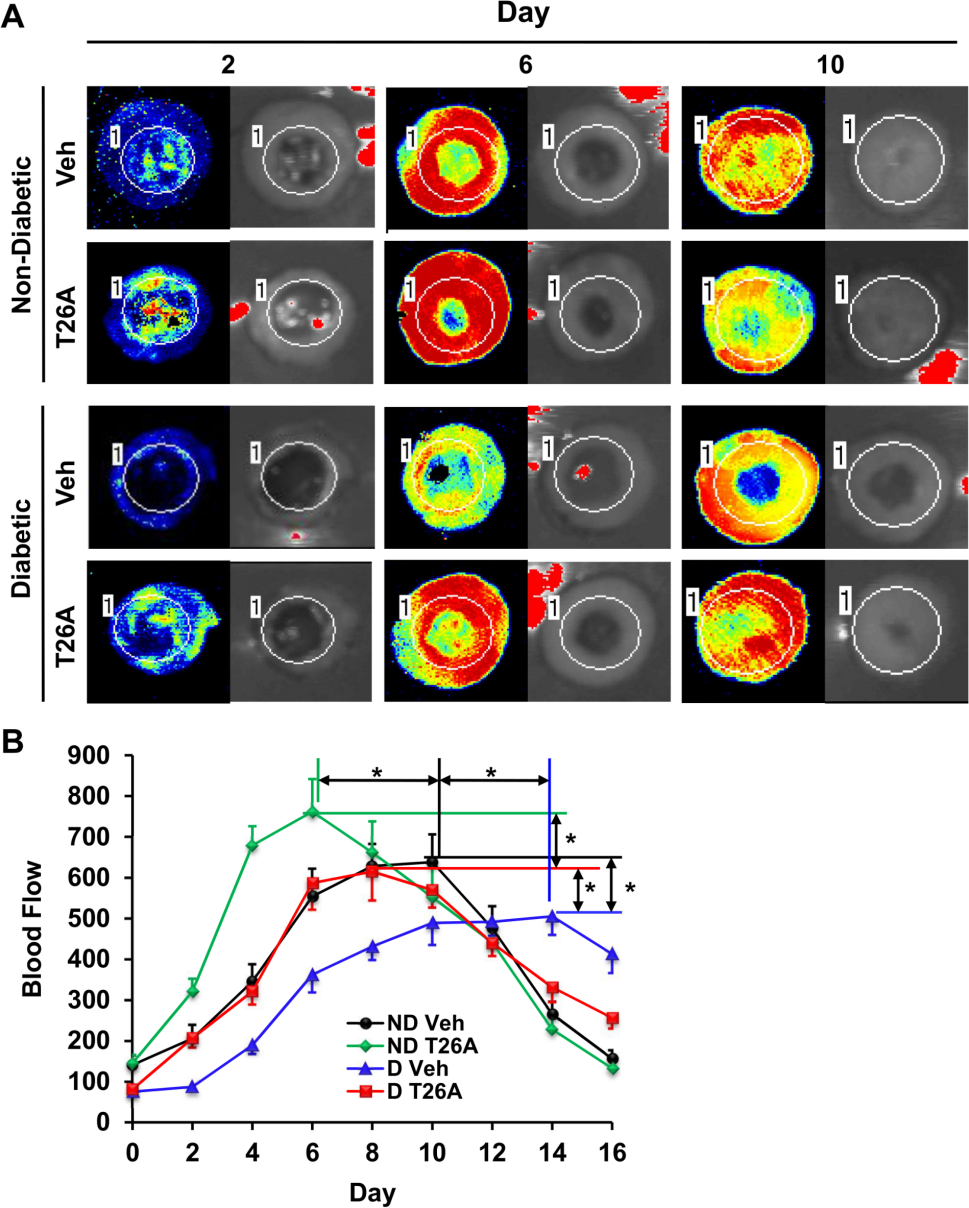
Inhibition of PGT Increases Perfusion to Cutaneous Wounds. (A) Representative images of blood flow in 10-mm wounds on the dorsa of non-diabetic (ND) Sprague Dawley rats or STZ diabetic (D) rats. Rats were treated with both i.p. injected 500 μL of vehicle (2% DMSO + 2% cremophor in water) or 1.2 mM T26A, one dose daily, and topically applied 30 μL of vehicle or 2 mM T26A, once every other day. Blood flow in the wound area was measured using a PeriScan PIM 3 immediately after wounding and every other day before fresh vehicle or T26A application. (B) Analysis of average blood flow in wounds during healing as a function of time. The color scale for Doppler measurements was set at 0–800, and the intensity was set at 0.34. Values are average ± SEM (n = 5). *p < 0.05, p values were obtained by ANOVA test.

To determine whether the increase in vessels due to T26A-induced neovascularization (shown in Fig 4A) had functional implications, we created 10-mm full-thickness wounds on the dorsa of rats and measured blood flow at wound sites immediately after wounding and every other day thereafter. Blood flow dropped to a low level after wounding. It gradually increased as the wounds healed, reaching a peak level and returning to the basal level thereafter (Fig 8). At day 2 there was slight increase in cutaneous blood flow in vehicle treated non-diabetic control wounds. However, T26A doubled blood flow in wounds compared to vehicle. Blood flow reached a maximum in T26A-treated wounds at day 6 (Fig 8B), whereas it took more than 10 days for blood flow to reach maximum in vehicle-treated non-diabetic wounds (Fig 8B). Vehicle- and T26A-treated wounds were 80% healed at days 10 and 6 (Fig 3A and 3B), respectively, which correlated with the days at which blood flow in the wounds peaked. Thus, local blood flow correlates with wound closure.

In diabetic wounds, there was no change in blood flow at day 2 (Fig 8), which is consistent with the lack of vascularization at day 2 (Fig 4). In diabetic rats it took 14 days for blood flow to reach peak level and peak flow was only about 70% of that measured in non-diabetic wounds (Fig 8). Whereas vehicle did not significantly affect blood flow in diabetic wounds, T26A not only increased peak flow, but also left-shifted the time course toward that of non-diabetic control wounds (Fig 8B). These results demonstrate that T26A-induced vessels were functional and contributed to accelerated wound healing.

## Discussion

Peripheral ischemia has a direct adverse impact on wound healing [34]. It is strongly associated with diabetes [30] and 46% of amputations in diabetic patients can be attributed to ischemia [35]. In the present study, by using diabetic rats and their non-diabetic matched controls, we identified a novel modulator of perfusion, PGT, and tested inhibition of PGT as an innovative strategy to mitigate peripheral ischemia and correct defective wound healing in diabetes. We found that inhibition of PGT increased arterial blood flow, promoted perfusion of peripheral tissues, enhanced migration of EPC and HEK, and stimulated neovascularization and re-epithelialization in cutaneous wounds, resulting in accelerated wound healing not only in non-diabetic rats, but more importantly, in diabetic rats.

Perturbed prostanoid lipid profiles have been reported in humans and rodents with diabetes mellitus. A reduced ratio of vasodilatory PGI_2_ to vasoconstrictive thromboxane (TxA_2_) has been reported in humans [36,37], which is a critical contributor to peripheral ischemia. Low PGE_2_ and or PGI_2_ were found in embryo, nerve and urine of diabetic rats [31,38,39]. Here for the first time we show that PGE_2_ level in blood of diabetic rat is only 30% that of non-diabetic rats (Fig 2F). In diabetic mice, we and others have shown that PGE_2_ is low in cutaneous wounds [18,40].

While the upstream common synthases (COX1 and COX2) of vasodilatory PGs and vasoconstrictory TxA_2_ are not altered in diabetic rodents or humans [40,41], we have found that the transporter that mediates the metabolism / degradation of PGs, PGT, is drastically induced by hyperglycemia in cultured dermal endothelial cells and in the skin of diabetic mice [18] and rats (S2 Fig), strongly suggesting that it is the induced PGT-mediated PGE_2_ degradation, rather than PGE_2_ biosynthesis, that is responsible for low PGE_2_ in diabetes. Systemic inhibition of PGT by i.v. T26A raises PGE_2_ levels in the circulation of both non-diabetic and diabetic rats (Fig 2F). Topically applied T26A increases PGE_2_ in cutaneous wounds of diabetic mice [18]. Thus inhibition of PGT can recover PGE_2_ and possibly other PGs in diabetes.

PGE_2_ and PGI_2_ are potent vasodilators. As degradation of TxA_2_ (a potent vasoconstrictor, product of COX1 and COX2) does not require PGT mediated process [42], the induced PGT selectively reduces vasodilatory PGs. It is conceivable that inhibition of PGT would cause vasodilation. Indeed, in a separate study we found that T26A potentiated PGE_2_ induced vasodilation of mouse aorta and reduced blood pressure in both mice and rats [43]. The vasodilatory effect of T26A could be a significant contributor to increased perfusion in hind limb. Under normal condition, PGs play a major role in controlling blood flow through large vessels [15]. When vascular occlusion occurs, PG synthesis and signaling are augmented, apparently in an attempt to maintain vasodilation and flow. Under diabetic condition, peripheral arteries are at high risk of being occlusive, limiting blood flow to distal tissues [5–8], as we show in this report that the blood flow in hind limb of diabetic rat is only 50% that of non-diabetic rats (Fig 1). However, applied exogenous PGE_2_ is able to increase blood flow (Fig 2D and 2E), indicating that vasodilatory role for PGs is maintained in diabetes mellitus, and suggesting a strategy wherein inhibition of PG metabolism, and thus raising the levels of endogenous PGs, would be beneficial for tissue perfusion in diabetes. Indeed, an inhibitor of PGT was able to enhance perfusion of intact and occluded hind limbs of diabetic rats (Fig 2D and 2E) by increasing PGE_2_ in the circulation (Fig 2F).

Note that increased blood flow from circulation to distal hind limb was a result of systemic inhibition of PGT by i.v. T26A (Fig 2A–2E), rather than local application of T26A. PGT is expressed in major organs and tissues including lung, heart, kidney, skeletal muscle and skin [18,44], and in several cell types such as endothelial and epithelial cells [18,45]. While systemically administered T26A can effectively increase perfusion in the hind limb, it is not impossible for locally applied T26A to increase blood flow in the hind limb, as hind limb is composed of skin, skeletal muscle, vasculatures, bone, epithelial cells and endothelial cells.

In addition to vasodilation, which facilitates movements of cells in the circulation, inhibition of PGT directly enhances the mobility of endothelial cells and epidermal keratinocytes (Figs 5, 7A and 7B). The vessels at the wound site shown in Fig 4A were marked by CD34 staining. CD34+ cells are bone-marrow derived progenitor cells, which are capable of differentiating into both endothelial and osteogenic lineages under the appropriate stimulating conditions [46]. Upon wounding the differentiation of circulating CD34+cells is directed towards the endothelial lineage [47] and endothelial cells migrate to the wound site to increase capillary density and stimulate neovascularization [48]. Inhibition of PGT increases vessels marked by CD34 at the wound site (Fig 4), probably by stimulating differentiation of CD34+ cells towards endothelial cells and by enhancing the mobility of CD34+ cells. The latter is evidenced by our *in vitro* data showing that either silencing or inhibiting PGT enhances migration of primary CD34+ cells freshly isolated from human bone marrow (Fig 5).

The effect of inhibition of PGT on migration of epidermal keratinocytes has been shown in our previous study [18]. Here we confirm that PGT directly regulates epidermal keratinocytes migration by utilizing siRNA technology (Fig 7A and 7B). To eliminate the concern that cells in the gap 12 hours after gap creation could be a result of proliferation, we chose 12 hours, much shorter than the doubling time of HEKs, which is about 26 hours [49]. During that 12 hours, neither inhibition nor silence of PGT had significant effects on HEK proliferation (data not shown). Another reason why gap closure was not due to proliferation is that the gap was created when cells were 100% confluent and PGE_2_ has no significant effects on proliferation of confluent keratinocyte culture [32]. Similar arguments could be applied to the migration assay of CD34+ cells. In case of CD34+ cells, we applied an additional assay, transwell assay. In the transwell assay, migrated cells from one side of the filter to the other during 8 hours should be solely attributed to migration, not proliferation. Together, these results indicate that PGT directly regulates migration of both HEKs and endothelial progenitor cells.

Although suppression of PGT did not significantly affect the proliferation of confluent keratinocytes, it did increase proliferation when keratinocytes were seeded at low density (Fig 7C). Together, the effects of PGT suppression on migration and proliferation of cultured keratinocytes support the *in vivo* effects of PGT inhibition on re-epithelialization.

So far we have shown that inhibition of PGT directly increases migration in human dermal microvascular endothelial cells [18], human endothelial progenitor cells (Fig 5), and human epidermal keratinocytes (Fig 7A and 7B). Together, these *in vitro* data obtained in human cells not only indicate that PGT directly regulates cell mobility, but also suggest that accelerated wound healing in rodents by inhibition of PGT can be potentially translated into humans.

Increased CD34+ cells resulting from inhibition of PGT would cause enhanced vasculogenesis, which is one of the two vascularization processes. The other process is called angiogenesis. We have reported that inhibition of PGT increases angiogenesis via induction of VEGF [18]. By stimulating both vasculogenesis and angiogenesis, inhibition of PGT enhances vascularization. Furthermore, the greater amount of new vessels formed as a result of T26A treatment are functional, as indicated by the higher level of blood flow at the wound site in T26A treated animals (Fig 8). Notice that the time for blood flow to reach peak level (Fig 8) is 1–2 days later than the time for the amount of vessels to reach peak level (Fig 4). This is because newly formed vessels need time to reorganize and then become functional.

Elevated blood flow at wound sites allows for the delivery of more nutrients and signaling mediators important to wound healing. Among those molecular mediators are growth factors, such as VEGF and platelet derived growth factor (PDGF), and hormone lipids such as PGE_2_ and PGI_2_. We have reported that local inhibition of PGT increases PGE_2_ and VEFG in cutaneous wounds in mice [18]. Here we show that systemic inhibition of PGT raises PGE_2_ in the circulation in rats (Fig 2F). Together these data provide a molecular mechanism by which PGT inhibition stimulates vascularization and accelerated wound healing.

In summary, we report a novel role of PGT in modulation of hind limb perfusion and in the mobilization of EPCs. Inhibition of the PG reuptake transporter PGT alone, or in combination with exogenous PGE_2_, appears to be a promising new approach to large vessel occlusion generally, and to wound healing specifically, especially as these two processes are altered pathologically in diabetes mellitus.

## Supporting Information

S1 Fig Experimental design. (A) Design for testing the effects of PGE_2_ and or PGT inhibitor, T26A, on peripheral perfusion. Hind limb ischemia was created by partial occlusion. Blood flow was measured before occlusion (BO) and after occlusion (AO). Either vehicle (Veh), PGE_2_ or T26A was injected via jugular vein after AO and blood flow was measured after injections. (B) Design for testing the effects of PGE_2_ and or T26A on cutaneous wound healing. Cutaneous wounds were created on the dorsa of rats. Intraperitoneal (i.p.) and or topical (Top) T26A or Veh was applied immediately after wounding. Thereafter, i.p. T26A or Veh was administered once daily until wounds closed. Top T26A or Veh was administered once every other day until wounds closed.
(DOCX)

S2 Fig PGT is induced in skin of diabetic rats. PGT mRNA expression levels in skin of non-diabetic Sprague Dawley and STZ induced diabetic rats (n = 5 per group), Values are average ± sd. **p < 0.01 by t-test. Total RNA was extracted from skin of rats with Trizol. 1μg of total RNA was used to synthesize cDNA with RTIIIase and OligodT from Life Technologies. Quantitative real time PCR using the Sybrgreen master mix was performed by a 7900HT PCR machine from Applied Biosystems. PGT (rat) primers: 5’TTTATGGCCTCCTCATCGAC3' (forward) and 5'CTGCAGGCTGTATTCCCTGT3' (backward). Beta-actin (rat) primers: 5'AAGTCCCTCACCCTCCCAAAAG3' (forward) and 5'AAGCAATGCTGTCACCTTCCC3' (backward).
(DOCX)
