# Supplementary material for: Inhibition of Prostaglandin Transporter (PGT) Promotes Perfusion and Vascularization and Accelerates Wound Healing in Non-Diabetic and Diabetic Rats
Source: PLoS One. 2015 Jul 31;10(7):e0133615. doi: 10.1371/journal.pone.0133615 (PMC4521828; doi:10.1371/journal.pone.0133615)
Supplement: S1 Fig — (A) Design for testing the effects of PGE2 and or PGT inhibitor, T26A, on peripheral perfusion. Hind limb ischemia was created by partial occlusion. Blood flow was measured before occlusion (BO) and after occlusion (AO). Either vehicle (Veh), PGE2 or T26A was injected via jugular vein after AO and blood flow was measured after injections. (B) Design for testing the effects of PGE2 and or T26A on cutaneous wound healing. Cutaneous wounds were created on the dorsa of rats. Intraperitoneal (i.p.) and or topical (Top) T26A or Veh was applied immediately after wounding. Thereafter, i.p. T26A or Veh was administered once daily until wounds closed. Top T26A or Veh was administered once every other day until wounds closed. (DOCX) [file pone.0133615.s001.docx]

S1 Fig. **Experimental design.** (A) Design for testing the effects of PGE_2_ and or PGT inhibitor, T26A, on peripheral perfusion. Hind limb ischemia was created by partial occlusion. Blood flow was measured before occlusion (BO) and after occlusion (AO). Either vehicle (Veh), PGE_2_ or T26A was injected via jugular vein after AO and blood flow was measured after injections. (B) Design for testing the effects of PGE_2_ and or T26A on cutaneous wound healing. Cutaneous wounds were created on the dorsa of rats. Intraperitoneal (i.p.) and or topical (Top) T26A or Veh was applied immediately after wounding. Thereafter, i.p. T26A or Veh was administered once daily until wounds closed. Top T26A or Veh was administered once every other day until wounds closed.
