# Supplementary material for: Inhibition of Prostaglandin Transporter (PGT) Promotes Perfusion and Vascularization and Accelerates Wound Healing in Non-Diabetic and Diabetic Rats
Source: PLoS One. 2015 Jul 31;10(7):e0133615. doi: 10.1371/journal.pone.0133615 (PMC4521828; doi:10.1371/journal.pone.0133615)
Supplement: S2 Fig — PGT mRNA expression levels in skin of non-diabetic Sprague Dawley and STZ induced diabetic rats (n = 5 per group), Values are average ± sd. **p < 0.01 by t-test. Total RNA was extracted from skin of rats with Trizol. 1μg of total RNA was used to synthesize cDNA with RTIIIase and OligodT from Life Technologies. Quantitative real time PCR using the Sybrgreen master mix was performed by a 7900HT PCR machine from Applied Biosystems. PGT (rat) primers: 5’TTTATGGCCTCCTCATCGAC3' (forward) and 5'CTGCAGGCTGTATTCCCTGT3' (backward). Beta-actin (rat) primers: 5'AAGTCCCTCACCCTCCCAAAAG3' (forward) and 5'AAGCAATGCTGTCACCTTCCC3' (backward). (DOCX) [file pone.0133615.s002.docx]

S2 Fig. **PGT is induced in skin of diabetic rats.** PGT mRNA expression levels in skin of non-diabetic Sprague Dawley and STZ induced diabetic rats (n = 5 per group), Values are average ± sd. **p < 0.01 by t-test. Total RNA was extracted from skin of rats with Trizol. 1µg of total RNA was used to synthesize cDNA with RTIIIase and OligodT from Life Technologies. Quantitative real time PCR using the Sybrgreen master mix was performed by a 7900HT PCR machine from Applied Biosystems. PGT (rat) primers: 5’TTTATGGCCTCCTCATCGAC3' (forward) and 5'CTGCAGGCTGTATTCCCTGT3' (backward). Beta-actin (rat) primers: 5'AAGTCCCTCACCCTCCCAAAAG3' (forward) and 5'AAGCAATGCTGTCACCTTCCC3' (backward).
